# Prevalent Ciliate Symbiosis on Copepods: High Genetic Diversity and Wide Distribution Detected Using Small Subunit Ribosomal RNA Gene

**DOI:** 10.1371/journal.pone.0044847

**Published:** 2012-09-14

**Authors:** Zhiling Guo, Sheng Liu, Simin Hu, Tao Li, Yousong Huang, Guangxing Liu, Huan Zhang, Senjie Lin

**Affiliations:** 1 Key Laboratory of Marine Bio-resources Sustainable Utilization, South China Sea Institute of Oceanology, Chinese Academy of Science, Guangzhou, Guangdong, China; 2 Department of Marine Sciences, University of Connecticut, Groton, Connecticut, United States of America; 3 Marine Biodiversity and Global Change Laboratory, Xiamen University, Xiamen, Fujian, China; 4 Department of Environmental Science, Ocean University of China, Qingdao, Shandong, China; University of North Carolina Wilmington, United States of America

## Abstract

Toward understanding the genetic diversity and distribution of copepod-associated symbiotic ciliates and the evolutionary relationships with their hosts in the marine environment, we developed a small subunit ribosomal RNA gene (18S rDNA)-based molecular method and investigated the genetic diversity and genotype distribution of the symbiotic ciliates on copepods. Of the 10 copepod species representing six families collected from six locations of Pacific and Atlantic Oceans, 9 were found to harbor ciliate symbionts. Phylogenetic analysis of the 391 ciliate 18S rDNA sequences obtained revealed seven groups (ribogroups), six (containing 99% of all the sequences) belonging to subclass Apostomatida, the other clustered with peritrich ciliate *Vorticella gracilis*. Among the Apostomatida groups, Group III were essentially identical to *Vampyrophrya pelagica*, and the other five groups represented the undocumented ciliates that were close to *Vampyrophrya/Gymnodinioides/Hyalophysa.* Group VI ciliates were found in all copepod species but one (*Calanus sinicus*), and were most abundant among all ciliate sequences obtained, indicating that they are the dominant symbiotic ciliates universally associated with copepods. In contrast, some ciliate sequences were found only in some of the copepods examined, suggesting the host selectivity and geographic differentiation of ciliates, which requires further verification by more extensive sampling. Our results reveal the wide occurrence and high genetic diversity of symbiotic ciliates on marine copepods and highlight the need to systematically investigate the host- and geography-based genetic differentiation and ecological roles of these ciliates globally.

## Introduction

Copepods are the most numerous metazoans distributed globally in the aquatic ecosystem [1]. They are conventionally considered as the key trophic linkage between phytoplankton and higher trophic levels in aquatic food chains [2], [3]. In addition, their chitinous skeleton can serve as a suitable living environment for the settlement and growth of various symbiotic microorganisms [4], [5]. Ciliates are one of the common protozoan symbionts found on copepods, of which subclasses Apostomatida, Peritrichia and Suctoria are the most common lineages [4], [6], [7]. The best documented ciliate symbionts on copepod are the apostome ciliates (subclass Apostomatida), a group of exuviotrophic/histophagous protozoans found on a wide variety of crustaceans including shallow- and deep-water copepods in both marine and fresh water [5], [8], [9]. The life styles of symbiotic apostome ciliates include ectocommensalism and invasive parasitism [4], [10]. They depend on copepod hosts to provide diverse and dynamic substrate and food sources to complete their life cycle [5]. Typical apostome genera include *Vampyrophrya*, *Gymnodinioides* and *Hyalophysa* [11], of which *Vampyrophrya* has been widely discovered to live as symbionts on copepod hosts around the world. For example, *V. pelagica* has been reported on *Acartia tonsa*, *A. longiremis*, *Centropages hamatus*, *C. typicus*, *Corycaeus* sp., *Eucalanus* sp., *Eurytemora* sp., *Labidocerca aestiva*, *Oncaea minuta* and *Paracalanus* sp. in North Carolina [12]. Hockin (1984) reported the occurrence of *V. pelagica* on several harpacticoid copepods from a sandy, intertidal beach in the river Ythan estuary, Aberdeenshire [13]. Peritrich and Suctorian ciliates have also been reported to live as epibionts on crustacean, including pelagic copepods [5], [7], [14], and they exhibit higher host-specificity on larger-sized hosts [5].

As copepods are abundant and widely distributed, the symbiotic ciliates on copepods have the potential to play an important role in affecting the ecological efficiency and carbon flux in the global ocean. The symbiotic apostomes mainly contain two groups. The first group, represented by *Gymnodinioides* and *Hyalophysa*, is exuviotrophic (feeding on exuvial fluid within the exoskeleton of the hosts), the other group, represented by *Vampyrophrya*, is histophagous, feeding on the tissues of hosts by entering the body through the wound created by their predators [2], [11], [15]. Although too many symbiotic ciliates on hosts will affect copepods’ moving and maintaining a desired depth [3], the exuviotrophs might benefit the hosts by decreasing predation pressure [16]. When hosts molt, the exuviotrophic apostome ciliates will sink to the deeper water together with the discarded molts, thereby playing an indispensable role in nutrient cycle [17]. However, previous reports also showed that the presence of peritrich ciliate *Epistylis daphniae* shortened the survival time of copepods in a food-deficient environment [18], [19]. The egg production rate, swimming efficiency and the ability of evading predators of copepods decreased when infested with peritrich ciliates [20], [21]. The histophagous apostome ciliates consume the host tissues, which will affect copepods directly by increasing their mortality, and the zooplanktonic predators of copepods indirectly by reducing the nutritive value of the copepods, thereby affect the higher trophic level [5].

Given their involvement in the process of material transfer in grazing and detritus food chains and potential threat to the well-being of the food web [2], it is very important to know whether these seemingly similar (based on morphology) symbiotic ciliates are genetically different and host- specific, so that their ecological roles can be further investigated and better understood.

So far, most of the research on marine copepod symbionts is limited to morphological observations of their presence using Chatton-Lwoff silver-impregnation technique or by scanning electron microscopy (SEM) and transmission electron microscopy (TEM) [2], [22]. However, the complex life cycles and difficulties in morphological observation make it difficult to determine the identities of the symbiotic ciliates; so far no study has been reported on the genetic diversity of copepod-associated apostome ciliates.

Molecular technique can be a powerful tool in addressing the genetic diversity of ciliates due to its sensitivity and specificity [23]–[25]. To date, information on genetic diversity of symbiotic ciliates is essentially lacking, with very few reports using molecular technique to study the phylogenetic positions of the symbiotic ciliates [26], [27]. In this study, we developed a PCR protocol using small subunit rRNA gene (18S rDNA) primer sets to PCR-amplify 18S rDNA from most of the eukaryotes yet exclude those of copepods, and applied it to investigate the genetic diversity and copepod host diversity of symbiotic ciliates in the Pacific and the Atlantic Oceans. To our knowledge, this is the first comprehensive molecular study of the ciliate assemblages on copepod hosts from a variety of geographic regions ranging from temperate to tropical oceans.

## Methods

### Copepod Collection

**Figure 1:**
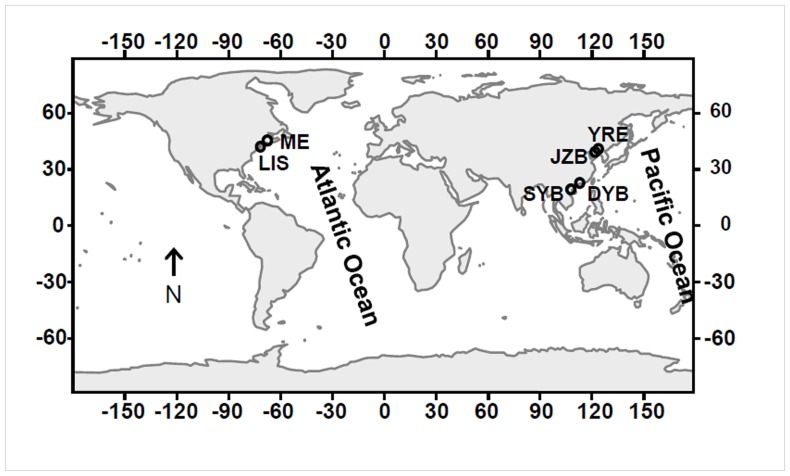
Sampling locations. LIS, Long Island Sound, Connecticut, USA; ME, Penobscot Bay, Maine, USA; SYB, Sanya Bay, China; DYB, Daya Bay, China; JZB, Jiaozhou Bay, China; YRE, Yellow River Estuary, China.

Copepod samples were collected using a plankton net (diameter 50 cm, mesh size 0.160 mm or 0.505 mm with a solid cod end) by gently towing ∼1–2 m below the surface for about 2 minutes. The contents of the net were transferred to a 10-L insulated container with natural seawater from the sampling location before being transferred to the lab for processing. Copepods were sampled from a wide range of geographic locations, including temperate regions (the Northwest Atlantic Ocean: Long Island Sound and Gulf of Maine, USA; Southern Yellow Sea: Jiaozhou Bay, China; Bohai Sea: Yellow River Esturary, China) and subtropical/tropical regions (South China Sea: Sanya Bay and Daya Bay, China) (Figure 1). No specific permits were required for the described field studies. Some live copepods were kept in a 1–L plastic container and transferred to the laboratory for gut clearance experiment (see next section). Plankton net-towed samples were immediately fixed in 2% Utermöhl’s solution [28] and transferred to the laboratory. Copepods were sorted and species was identified morphologically according to Chen and Zhang (1965) and Gerber (2000) under a stereomicroscope (Lecia, S8APO) [29], [30]. Utermöhl’s solution-fixed copepod samples were then stored at 4°C for a short period of time before DNA extraction (within 1 month), a storage condition without detectable DNA degradation in the samples [31].

### Copepod Gut Clearance

Live copepod individuals were brought directly to the laboratory and transferred into a 500-mL beaker containing 200-mL autoclaved 0.45µm-filtered seawater (FSW) supplied with diatom *Thalassiosira weissflogii* (at 10^4^ cells mL^−1^) as food for 3 days. The diatom was cultured in f/2 medium [salinity 33±1 practical salinity units (PSU), pH 8.0] at 25°C on a 12∶12 h light: dark cycle at ∼100 µmol photons m^–2^s^–1^. During the feeding period, every 6 hours the copepods were transferred to a piece of 10-µm mesh and rinsed 3–5 times with fresh FSW to clear off any attached detritus, and then transferred to fresh seawater enriched with *T. weissflogii* again to purge out any residual natural food from the gut. After this procedure was repeated for about 10 times, the copepods were transferred into FSW and kept for two more days to empty the gut content. The copepods were then preserved in 2% Utermöhl’s solution, sorted, and identified to species, and stored at 4°C before DNA extraction as mentioned above.

### DNA Extraction

The sorted copepods were serially rinsed five times with FSW followed by one final wash in autoclaved ultrapure water to remove salts, and then examined under a compound microscope to check if there were any visible organisms/debris attached on the surface. Five copepod individuals of the same species were combined and homogenized thoroughly in microfuge tubes using disposable micropestles and incubated with 0.5 ml DNA buffer (1% SDS, 100 mM EDTA pH8.0, 200 µg mL^−1^ proteinase K) at 55°C for 48 hours. DNA was extracted and purified essentially as described previously [32], and its quality was checked by PCR using a set of universal 18S rDNA primers [31], [32] and stored at −20°C.

### Primer Design, PCR, Cloning and Sequencing

We searched GenBank database for copepod 18S rDNA sequences and aligned them using CLUSTAL W (1.8) [33] with representatives of other eukaryotes reported in GenBank database (>100 lineages). Based on the alignment, new primer sets were designed on regions that were conserved in eukaryotes but unique in copepods. These primers were aimed to embrace the 18S rDNAs of as many lineages of eukaryotes as possible but to exclude those of copepods. Several primers were designed and tested (H. Zhang et al. unpubl.), and the set of Non-copepod 18SF2 (5′-AGCAGGCGCGHAAATTRCCCAATCY-3′) and Non-copepod 18SR2 (5′-CCGTGTTGAGTCAAATTAAGCCG-3′), with a corresponding PCR amplicon of ∼0.8 kb in length, was chosen in this study. Copepod DNA samples from starved as well as in situ fixed copepods were used as the templates for PCR using the following touch-down program: an initial denaturing step at 94°C for 1 min, 5 cycles of denaturing at 95°C for 30 s, annealing at 68°C for 30 s, and extension at 72°C for 40 s, followed by 30 cycles of denaturing at 95°C for 10 s, annealing at 62°C for 30 s, and extension at 72°C for 40 s, and an final elongation step of 7 min at 72°C. PCR products were purified and cloned; more than 50 clones for in situ sample were randomly picked and sequenced as reported [34].

### Phylogenetic Analysis

Sequences obtained were searched against GenBank database using the Basic Local Alignment Search Tool (BLAST). The best hits were aligned with the sequences obtained in this study using CLUSTAL W (1.8) [33]. If the top hit was of an unknown species, the closest hit with a species identity was recruited. The alignment dataset was run through ModelTest v3.7 [35] to identify the best-fit nucleotide substitution model. The best-fit model Tamura 3-parameter with gamma distribution (T92+G) was then employed for maximum likelihood analysis using PhyML package [36]. In addition, Neighbor Joining (NJ) analysis was also performed using the same program. The reliability of the tree topology was evaluated using bootstrap analysis with 1000 resampling for NJ analysis and 500 for ML analysis.

## Results

### Ciliate 18S rDNAs Detected from Both Starved and in situ Fixed Copepod Samples

Ten copepod species from six families (Calanidae, Eucalanidae, Paracalanidae, Temoridae, Centropagidae, Acartiidae) were identified based on morphological characteristics (Table 1). No obviously visible organisms/debris were found attached on the surface of copepods under the magnification used to screen hosts. Some live individuals of these copepods were used in gut clearance treatment, and three species (*Temora turbinata*, *Acartia erythraea* and *A. pacifica*, named as starved copepods) survived the treatment, which were subjected to DNA extraction, PCR and cloning. For *T. turbinata*, 28 resultant clones were sequenced and 27 of them shared the highest nucleotide identity (96–99%) to apostome ciliate *Vampyrophrya pelagica* 18S rDNA (EU503539). We also sequenced a few clones for *A. erythraea* and *A. pacifica*, and *V. pelagica*-like sequences were also detected (Table 1).

**Table 1:** Taxonomic and biological information of the copepods and 18S rDNA ciliate sequences number obtained.

Species	Family	Stage/Sex	T (°C)	S (PSU)	Sampling date	Station*	Symbiotic ciliate sequences
**Starved samples**							
*Acartia erythraea*	Acartiidae	Adult/female	28.7		24-May-11	SYB	1
*A. pacifica*	Acartiidae	Adult/female	28.7		24-May-11	SYB	1
*Temora turbinata*	Temoridae	Adult/female	28.7		24-May-11	SYB	27
**In situ fixed samples**							
*Centropage tenuiremis*	Centropagidae	Adult/female	28	32	11-Apr-11	DYB	80
*Paracalanus parvus*	Paracalanidae	C IV/	28	32	11-Apr-11	DYB	20
*A. tonsa*	Acartiidae	Adult/female			31-Oct-07	LIS	19
*A. tonsa*	Acartiidae	Adult/female			21-Oct-07	ME	50
*A. erythraea*	Acartiidae	Adult/female	28		24-May-11	SYB	26
*Canthocalanus pauper*	Calanidae	Adult/female			21-Jul-10	SYB	29
*Temora turbinata*	Temoridae	Adult/female			21-Jul-10	SYB	14
*A. pacifica*	Acartiidae	Adult/female	17	33.5	29-Oct-10	JZB^1^	29
*Centropage dorsispinatus*	Centropagidae	Adult/female	23	28	28-Sep-10	YRE	70
*Calanus sinicus*	Calanidae		16	32	14-Jun-10	JZB^2^	25
*Subeucalanus subcrassus*	Eucalanidae	Adult/female			21-Jul-10	SYB	0

* DYB, Daya Bay, China (114°32′41.5″E, 22°35′19.9″N); JZB, Jiaozhou Bay, China (^1^∶120°38′55.87″E, 36°19′04.53″N; ^2^∶120°20′52.01″E, 35°58′42.05″N); SYB, Sanya Bay, China (109°25′29.94″E, 18°11′59.94″N); YRE, Yellow River Estuary, China (119°31′34.44″E,37°41′16.32″N); LIS, Long Island Sound, Connecticut, USA (72°03′48.6′′W, 41°18′55″N); ME, Penobscot Bay, Maine, USA (68°41′1.10′′W, 44°20′48.28′′N).

**Figure 2:**
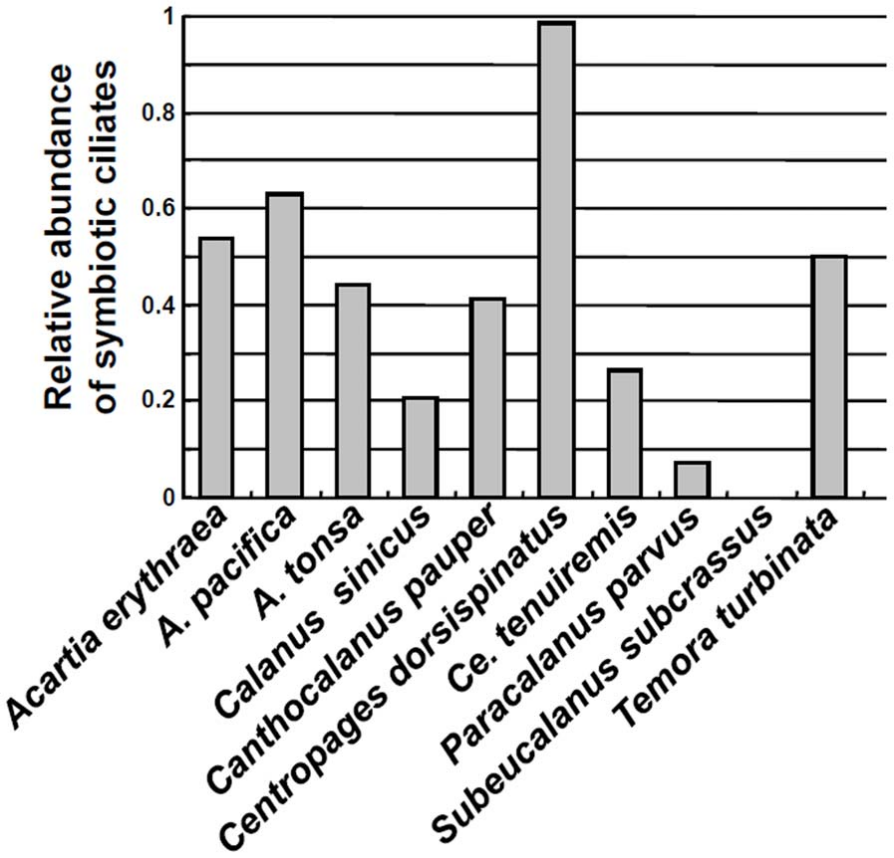
Ratio of symbiotic ciliate groups. Relative abundances of symbiotic ciliate (Apostomatida and related) 18S rDNA groups in all sequences obtained from the in situ fixed copepods (*Acartia erythraea*, *A. pacifica*, *A. tonsa*, *Calanus sinicus*, *Canthocalanus pauper*, *Centropages dorsispinatus*, *C. tenuiremis*, *Paracalanus parvus*, *Subeucalanus subcrassus*, *Temora turbinata*).

DNA samples isolated from the 11 in situ copepod samples (i.e. containing food in the gut) of those10 species (Table 1) were also subjected to PCR amplification. In addition to various other eukaryotic sequences derived from copepod diets (S. Lin and H. Zhang unpubl.), ciliate sequences were detected from all but one (*Subeucalanus subcrassus*) samples. Several ciliate sequences obtained from *Acartia tonsa* in Long Island Sound and Maine were of planktonic ciliate species (S. Lin and H. Zhang unpubl.), but all the rest were closest (88–99%) to the *V. pelagica*-like sequences found from the starved copepods described above or other known symbiotic ciliates, but distant (>20% difference) from other organisms and were hence considered as symbiotic ciliate sequences here. Totally, 362 symbiotic ciliate sequences were obtained from all the in situ fixed copepod samples, 357 of which were close to those of apostome ciliates *Vampyrophrya/Gymnodinioides/Hyalophysa*; the remaining five were close to that of the peritrich ciliate *Vorticella gracilis* (GQ872429) (for details see next section). Among the 10 species of copepod hosts fixed in situ, *A. erythraea*, *A. pacifica*, *Centropages dorsispinatus*, and *T. turbinata* appeared to harbor higher abundances of symbiotic ciliates, because the 18S rDNA sequences of symbiotic ciliates accounted for over 50% of the total non-copepod eukaryotic 18S rDNA sequences obtained, while the relative abundance of symbiotic ciliate 18S rDNA in the other 6 copepod species ranged from 7–43% (Figure 2).

### General Phylogenetic Relationships of the Ciliate 18S rDNA Sequences

**Figure 3:**
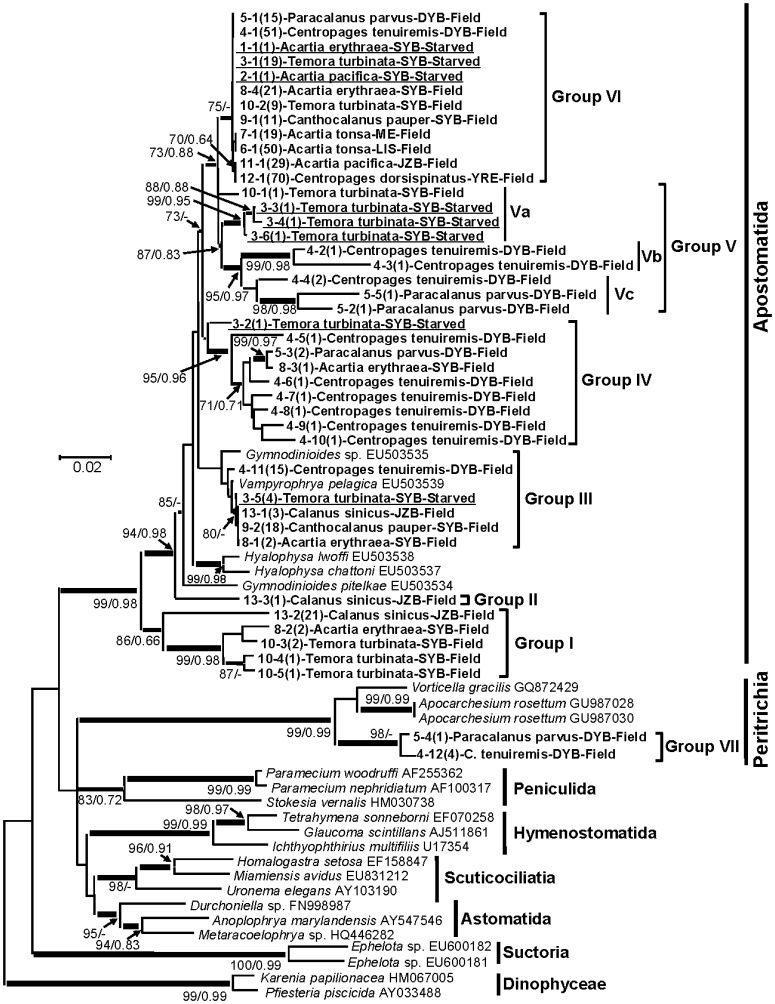
Neighbor joining and maximum likelihood analyses of 18S rDNA sequences. Phylogenetic tree inferred from partial 18S rDNA sequences retrieved in this study and the related organisms available in GenBank. Shown at nodes are bootstrap value from Neighbor-Joining (left) and SH-like value of maximum likelihood (right) trees; only values >70%/0.6 at critical nodes are shown. The thickest branches denote bootstrap values of >90%, medium-thick branches values of 70 to 90%, and thin branches values of <70%. The numbers at nodes are bootstrap confidence values based on 1000 replicates for NJ analysis and 500 for ML analysis, while the numbers in parentheses indicate number of identical sequences obtained. The scale bar indicates the substitutions rate per nucleotide. LIS, Long Island Sound, Connecticut, USA; ME, Penobscot Bay, Maine, USA; DYB, Daya Bay, China; SYB, Sanya Bay, China; JZB, Jiaozhou Bay, China; YRE, Yellow River Estuary, China. “Starved” denotes the sequences retrieved from gut-purged starved copepods, while “Field” from in situ fixed copepod samples.

The 391 ciliate 18S rDNA sequences obtained were clustered to collapse redundancy, yielding 43 unique sequences. These sequences were aligned with those of the representatives from the major orders (or subclasses) of class Oligohymenophorea that were significant hits in the BLAST analysis of our obtained sequences. Two dinoflagellate 18S rDNA sequences were included as the outgroup. Both NJ and ML trees gave similar tree topologies (Figure 3). Most of the sequences obtained were grouped with apostome ciliates (Apostomatida) with moderate to strong support. These sequences were further clustered into six ribogroups (**Groups I-VI**). **Group I** (27 sequences) was basal to the other apostome sequences with strong support and relatively longer branches in the phylogenetic trees, and was closest to the amphipod-hosted ciliate *Gymnodinioides pitelkae*, suggesting that this group may be a member of another order in subclass Apostomatida. **Group II** consisted of only one sequence from *C. sinicus* close to *G*. *pitelkae* with strong NJ bootstrap support. **Group III** sequences were clustered with *V. pelagica* (>99% identity) with moderate bootstrap in both the NJ and ML trees. **Group IV** mainly consisted of sequences retrieved from copepods *C. tenuiremis* and *Paracalanus parvus* from Daya Bay, China with moderate to strong NJ and ML supports; these sequences shared 92–95% identity with *Vampyrophrya/Gymnodinioides/Hyalophysa*, indicating this may be a new group close to Foettingeriidae. **Group V** sequences were closest to *V. pelagica* (90–97% nucleotide identity). This group could be further divided into three subgroups: sequences from *T. turbinata* (**Va**), from *C. tenuiremis* (**Vb**), and from both *C. tenuiremis* and *P. parvus* (**Vc**), in which Vb and Vc contained more diverse sequences with longer branches in the trees. **Group VI** contained almost identical ciliate sequences from 8 copepod species collected from temperate regions of North Atlantic Ocean, and temperate to tropical regions of the western Pacific Ocean; all these sequences shared highest identity (97%) to *V. pelagica*. The five sequences from *C. tenuiremis* and *P. parvus* collected from Daya Bay, China, formed a separate group (**Group VII**) within subclass Peritrichia, and shared 95–96% identity to *Vorticella gracilis*.

### The Associations of Symbiotic Ciliates with different Copepod Species and Geographic Locations

**Figure 4:**
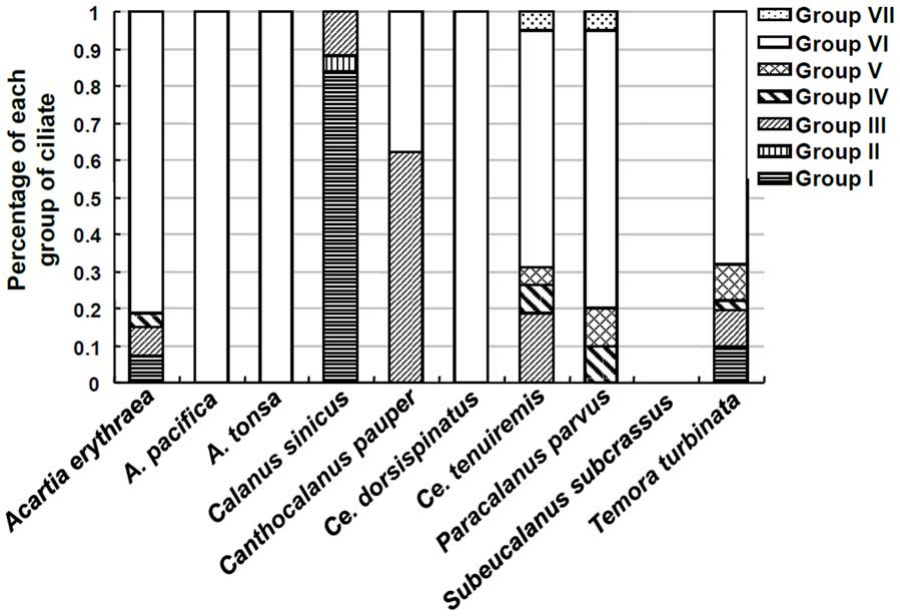
Relative abundance of each ribogroup of ciliate symbionts out of total symbiotic ciliate sequences. Fraction was calculated as the sequence number of each ribogroup divided by the total number of symbiotic ciliate sequences obtained from each copepod species. Data from starved copepods were not included in this figure because the total number of clones sequenced for each species was generally low precluding the possibility to achieve reliable fraction values.

**GroupVI** sequences were most abundant among all symbiotic ciliate sequences obtained. They were detected in 8 different copepod species and occupied >50% of the total ciliate sequences obtained from the copepod in most of the cases (Figure 4). **Groups I**, **III**, **IV**, **V** and **VII** ciliates were also found in 3 to 5 different copepods hosts, suggesting that these ciliates were not restricted to a specific host.

Of the ten copepods species fixed on-site, three (*A. pacifica*, *A*. *tonsa*, *C*. *dorsispinatus*) were infested with only one group of ciliates, mainly from **GroupVI** (Figures 3, 4). Four copepod species (*A. erythraea*, *C. tenuiremis*, *P. parvus*, *T. turbinata*) each harbored 4–5 different groups of ciliates. Therefore, some copepod hosts were infested with different genotypes of ciliates while other hosts were infested with fewer types.

Our data also showed some geographic pattern of the ciliate groups. **Group II** ciliates were only detected from the copepod samples in JZB (Qingdao), the temperate region in the Southern Yellow Sea (North Pacific), while **Groups IV** and **V** only in DYB and SYB, the tropical/subtropic regions in the South China Sea (North Pacific). On the contrary, **Group VI** ciliates were found in all sampling sites ranging from temperate to tropical waters and from both the Atlantic and the Pacific. **Groups I** and **III** also occurred in both temperate and tropical oceans, but only in the Pacific.

## Discussion

Most of the previous studies on marine copepod symbionts were carried out using morphological observations by silver-impregnation technique or SEM and TEM, which are time-consuming and require specific equipment, and were limited to the equipped laboratories [2], [22], [25]. These studies have concentrated on description of symbiotic ciliate life cycle and cell anatomy ([5] and refs therein) rather than species abundance and diversity. No study has been reported on apostome ciliate species diversity. In this study, we developed a primer set which could PCR-amplify 18S rDNA from most of the non-copepod eukaryotes, and applied it to study genetic diversity of ciliate symbionts on copepods from several geographic regions. Among all possible gene markers, we chose to use 18S rDNA because this gene has been sequenced from most lineages of eukaryotes, and it contains conserved and variable regions that facilitate primer design. It is also the largest database among ciliate genes (2786 sequences on February 8, 2012) including sequences of all the major groups of ciliates (e.g. Figure 3); this offers good chance of identifying ciliate species from the sequences obtained and facilitates the study of genetic diversity and species diversity on symbiotic ciliates. The Non-copepod 18SF2-R2 primer set amplifies a ∼800 bp DNA fragment, covering 43% of 18S rDNA. This sequence contains three variable regions (V3, V4, V5), of which V4 is highly variable, and has been used to reveal the diversity of a eukaryotic community [37] or that of a specific group [38] in coastal waters. The sequences we obtained were closest (88–99% identical) to 18S rDNA of various known ciliate symbionts, and quite distant (>20% difference) from other organisms. This result indicates that the 18S rDNA region we used in this study is a promising DNA marker to identify symbiotic ciliates on copepods. Using traditional morphological identification method, Valbonesi and Guglielmo [39] and Ohtsuka et al. [2] investigated different copepod species and found that 75% to 84% of them were infected by symbiotic ciliates. In this study, we found higher infection rate (90%) among all the copepod species obtained. While morphology-based studies suggest that some lineages of the symbiotic ciliates (e.g. *Vampyrophrya*) are associated with copepods around the world [2], the employment of this gene in this study however showed existence of distinct genotypes of the detected ciliates close to yet distinct from *V. pelagica*, demonstrating that molecular technique is powerful in addressing the symbiotic ciliate genetic diversity due to its sensitivity and specificity and will be useful for more systematically investigating host and geographic specificity of these ciliates.

### Prevalence and Wide Distribution of Ciliate Symbionts on Copepods

This study is the first attempt to systematically investigate occurrence of ciliate symbionts on a variety of copepods collected from different geographic locations using molecular method. Symbiotic ciliates were detected successfully on all but one copepod samples. Geographically, these copepod samples covered from tropical to temperate in the Pacific Ocean and two distinct temperate marine environments in the Atlantic Ocean. Of the 391ciliate sequences obtained, 386 were close to *Vampyrophrya/Gymnodinioides/Hyalophysa*, apostome ciliates known to be crustacean symbionts; the other 5 sequences showed the highest identity to the peritrich ciliate *Vorticella gracilis*, a member of the *Vorticella* genus known to live on copepods [40].

To avoid the “noise” of diets remaining in the guts of the copepods while achieving the ciliate symbiont sequences, we used *T. weissflogii*, a diatom suitable as diet for copepods, to purge the gut contents [41], [42], then starved the copepods before isolating DNA from the copepod samples. By these procedures, the ciliate 18S rDNA sequences subsequently derived from the starved copepods should not have come from the natural diet that copepods had ingested in the sea. For the starved *T. turbinata* samples, 27 apostome ciliate sequences were clustered into four groups, indicating this species harbored a high diversity of ciliates. We also examined the in situ fixed copepod samples including this species. In addition to the highly diverse 18S rDNA sequences from ingested food (S. Lin and H. Zhang unpubl.), sequences of apostome ciliates were also detected from the in situ fixed copepods (Figure 3). These sequences were highly similar to those found in the starved copepods, indicating these ciliates from the in-situ fixed copepods were also symbiotic ciliates rather than ingested ones. Interestingly, besides the apostome ciliates, 5 sequences similar to peritrich ciliates known to be symbiotic were also obtained from the in situ fixed copepods. The peritrich ciliates were not detected from the starved copepods, likely due to the long copepod gut clearance process (washing, starving, holding in seawater for a few hours) causing many peritrich and/or suctoria ciliates to detach and leave their hosts, while the apostome ciliates would remain in an encysted form. However, out of 362 ciliate sequences obtained from in situ fixed copepod samples, 357 were of apostome ciliates, indicating the apostome ciliates were the main ciliate symbionts of the copepods studied.

The apostome and peritrich ciliates have been reported to be symbionts of a wide variety of copepods all over the world (e.g., [2], [38], [41]), and most of the copepod species we analyzed in this study have been reported to be infected with symbiotic ciliates. Ohtsuka et al. (2004) has observed *V. pelagica* on various copepod hosts in the Seto Inland Sea, Japan, including *P. parvus*, *A. tonsa*, *A. pacifica*, *C. tenuiremis*, *S. subcrassus*, and *C. sinicus* by SEM and TEM [2]. As symbiotic ciliate 18S rDNA sequences were detected in most of the copepod samples examined, it is quite clear that symbiotic ciliates are prevalent on copepods (Figure 2). Moreover, out of the 10 in situ fixed copepod species, higher percentage of symbiotic ciliate 18S rDNA sequences were obtained from *A. erythraea*, *A. pacifica*, *C. dorsispinatus*, and *T. turbinata*, suggesting that these copepods might harbor somewhat higher abundances of symbiotic ciliates than other lineages of copepods (Figure 2). These taxa may contribute more to the biogeochemical process of carbon flux caused by copepod-associated symbiosis as they are common marine copepods. Biases due to high copy numbers of 18S rDNA in ciliates [43] can contribute to the dominance of ciliate 18S rDNA sequences in a mixed eukaryote assembly; however, this does not seem to be the reason for the dominance of the symbiotic ciliates in this study, because in some of these copepod samples (*A. tonsa* from Long Island Sound and Maine), planktonic ciliates known to be food of copepods were also detected, but in much lower abundances (S. Lin and H. Zhang unpubl.). Real-time quantitative PCR should be conducted in future studies to quantify the abundance of these ciliates. No ciliate sequences were obtained from *S. subcrassus* DNA, in contrast to the result by Ohtsukaet al. (2004), which showed presence of ciliate symbionts on this copepod. This may be due to the real absence of ciliate symbionts on *S. subcrassus* samples in this study, or inadequate sequencing depth.

### High Diversity of Ciliate Symbionts on Copepods

As indicated above, most of the 391 ciliate sequences obtained were grouped with apostome ciliates, close to *V. pelagica*, which would probably only be grouped to few morphotypes microscopically due to the complex life cycles of apostome ciliates and the difficulties in morphological observation. However, the molecular analysis revealed that these apostome ciliate assemblages were diverse. These sequences could be divided into six distinct groups. Except **Group III** that appeared to be *V. pelagica*, other five groups represented distinct genotypes (or even multiple species) of previously undocumented symbiotic ciliates, as there is no current "cutoff" that can be used to interpret morphological differences (and species criteria) with sequence differences. The diversity of ciliate symbionts differed among copepod host species. *A. erythraea*, *C. tenuiremis*, *P. parvus* and *T. turbinata* were each infested with different (4–5) groups of ciliates, while other hosts (*A. pacifica*, *A. tonsa*, *C. dorsispinatus*) were only infested with a particular group of ciliates (**Group IV**) (Figure 4). We also detected peritrich ciliates on *C. tenuiremis* and *P. parvus*. Peritrich ciliates, a large and distinctive ciliate lineage, are widespread epibionts on copepods in freshwater and marine environments. For example, copepod *Eucyclops agilis* has been found to be infested with peritrich ciliate *Epistylis plicatilis* [44], *C. abdominalis* with *Zoothamnium* [45], *Thermocyclops decipiens* with *Rhabdostyla* sp. and *Scyphidia* sp. [46]. Peritrich ciliates have not been found on other copepods in our study, probably because they are host- and stage-specific [5], and the settlement of peritrichs has a rigid requirement for substrate surfaces [47].

Suctorian ciliates of class Phyllopharyngea have been reported to live as the epibionts on crustaceans especially pelagic copepods [3], [5], [48], and they exhibit higher host-specificity on hosts of larger size and greater longevity [5]. However, no suctorian ciliates were detected in this study, which may be attributed to high host-specificity, unsuitable environment, or insufficient sequencing depth.

### Universal 18S rDNA Genotype

Among the ciliate sequences obtained, **Group V**I ciliates were the most abundant and ubiquitous, having been found in all but one species (*C. sinicus*) of copepods collected from all the different locations in this study (Figures 3, 4). This result agrees with the previous morphological observation of apostome ciliate *V. pelagica* infecting various pelagic copepods in the Seto Inland Sea, Japan [2], but expands the distribution of this lineage of ciliates to different geographic locations. Moreover, our **Group VI** genotype showed only 97% identity to *V. pelagica* 18S rDNA as the closest hit in BLAST, suggesting this may be a distinct undocumented species in this genus. As such, this result implies that there may be multiple *Vampyrophrya* genotypes (or species) that are cosmopolitan in association with copepods.

### Potential Importance of Symbiotic Ciliates in Marine Biogeochemical Processes

While planktonic ciliates are generally important in consuming phytoplankton and recycling the nutrients in aquatic environment, symbiotic ciliates heavily living on the abundant copepods can also be important players in the food chain [5]. Besides copepods, some of these symbiotic ciliates also infest euphausiacean and other planktonic crustaceans [49], potentially with wider distribution and greater ecological roles than recognized on the copepods. Therefore, the role of the symbiotic ciliates in the aquatic food web should not be overlooked, and further systematic studies are needed to assess the magnitude of the contribution of these symbionts to carbon flux and the biology of copepod in the global ocean. The PCR protocol based on the unique 18S rDNA primer set we developed in this study will facilitate such studies. In addition, 18S rDNA sequence data of the symbiotic ciliates from the present study will be valuable addition to the GenBank database, which still needs to be substantially augmented to facilitate rapid identification of ciliate symbionts.

The ciliate 18S rDNA sequence data have been deposited at GenBank under accession numbers JX417888-JX417930.
